# Supplementary material for: The social costs of aviation CO2 and contrail cirrus
Source: Nat Commun. 2025 Sep 29;16:8558. doi: 10.1038/s41467-025-64355-5 (PMC12480469; doi:10.1038/s41467-025-64355-5)
Supplement: Supplementary file 1 — Supplementary Information [file 41467_2025_64355_MOESM1_ESM.pdf]

Supplementary information

The social costs of aviation CO_2 and contrail cirrus

Daniel J.A. Johansson[,1], Christian Azar[1], Susanne Pettersson[1], Thomas Sterner[2], Marc E.J. Stettler[3], Roger Teoh[3]*

[*]Corresponding author. E-mail: daniel.johansson@chalmers.se

[1]*Division of Physical Resource Theory, Department of Space, Earth and Environment, Chalmers University of Technology, Gothenburg, Sweden.*

[2] *Department of Economics, School of Business, Economics and Law, University of Gothenburg, Gothenburg, Sweden.*

[3] *Department of Civil and Environmental Engineering, Imperial College London, London, UK.*

Supplementary Note 1. Illustration of model approach

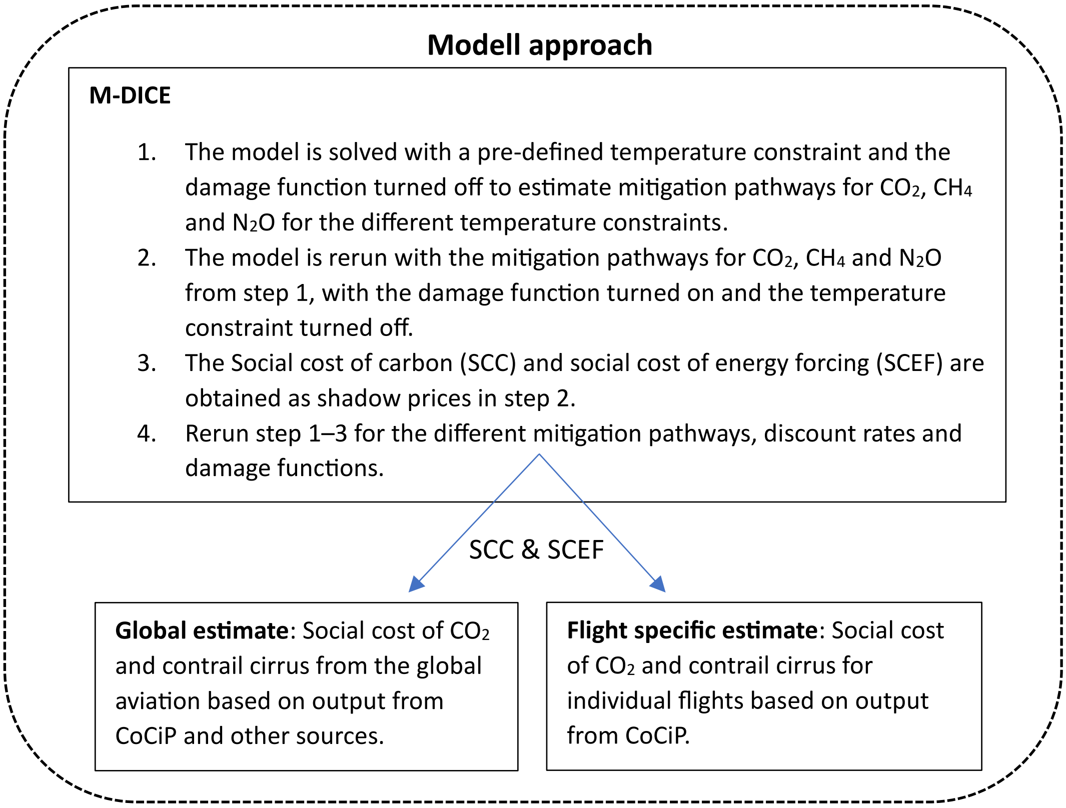

Supplementary Fig. 1. Modelling framework. Schematic illustration of the modelling approach used in the study.

Supplementary Note 2. Emission pathways for CO_2, CH_4 and N_2O

To estimate the social cost of CO_2, of energy forcing (EF) and of contrail cirrus, we include three pathways consistent with different climate stabilization levels (3°C, 2°C, and 1.5°C with overshoot) in the main part of the paper (in Supplementary Note 4 we analyze the consequences of two additional pathways stabilizing at 4°C and 1.0°C with overshoot). In Supplementary Fig. 2, we present pathways for temperature, CO_2, CH_4 and N_2O emissions generated by M-DICE for the five main cases. The emissions pathways are largely consistent with emission pathways generated by process-rich IAMs[1].

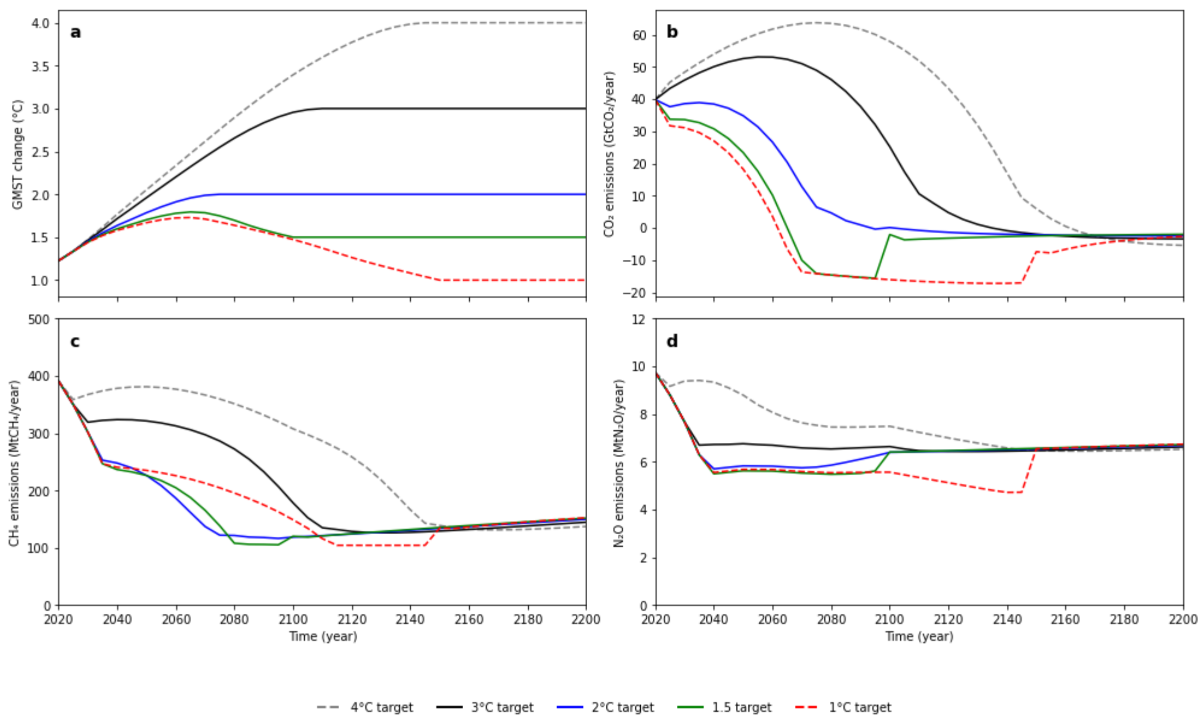

Supplementary Fig. 2. Temperature and emissions pathways. Surface temperature change pathways (a) and corresponding anthropogenic CO_2 (b), CH_4 (c) and N_2O (d) emissions pathways. Pathways are generated with M-DICE.

Supplementary Note 3. Uncertainty in contrail forcing for individual contrails

The uncertainty analysis in the paper is based on the use of ensemble members of the ERA5 reanalysis (HRES) dataset[2]. This ensemble is used as input to CoCiP to generate contrail cirrus energy forcing estimates. There are 10 ensemble members of the ERA5 available where each member is based on a realization of the output of the Integrated Forecasting System (IFS) for different assumptions on uncertain observations and model parameters. The uncertainty analysis is also based on an estimate of systematic uncertainties in the energy forcing calculations and the efficacy value for contrail cirrus energy forcing by using a probability density function (for the combined effect) estimated from the literature.

The EF per km for each flight for the 10 different outputs from CoCiP based on the 10 different ERA5 ensemble members is shown in Supplementary Fig. 3. The EF per km (y-axis) for each flight is shown for an ordering of the flights from the lowest to the highest EF per km in the base case run, i.e., based on the contrail implications of the control member in the ensemble, here called “run_000”. The ordering for the other runs, i.e., based on alternative ERA5 ensemble members, are kept the same, and the y-axis shows the resulting EF per km flight generated by CoCiP for the specific weather and radiation conditions for the specific ERA5 ensemble member. As seen in Supplementary Fig. 3 the contrail EF per km is sensitive to which ensemble member is used as input to CoCiP. For example, contrail cirrus EF that is negative for a specific flight when using the main ERA5 ensemble member may be negative, positive or zero when using another ensemble members as input to CoCiP.

In Supplementary Fig. 4 we show a hexbin plot with EF per km for each flight on the x-axis generated by CoCiP based on the main case ERA5 ensemble member (run “000”), while the EF per km generated by CoCiP for the other ensemble members are shown on the y-axis. From the figure it is

clear that there is strong correlation in the EF generated by the runs for the same flight, but there is also substantial variability.

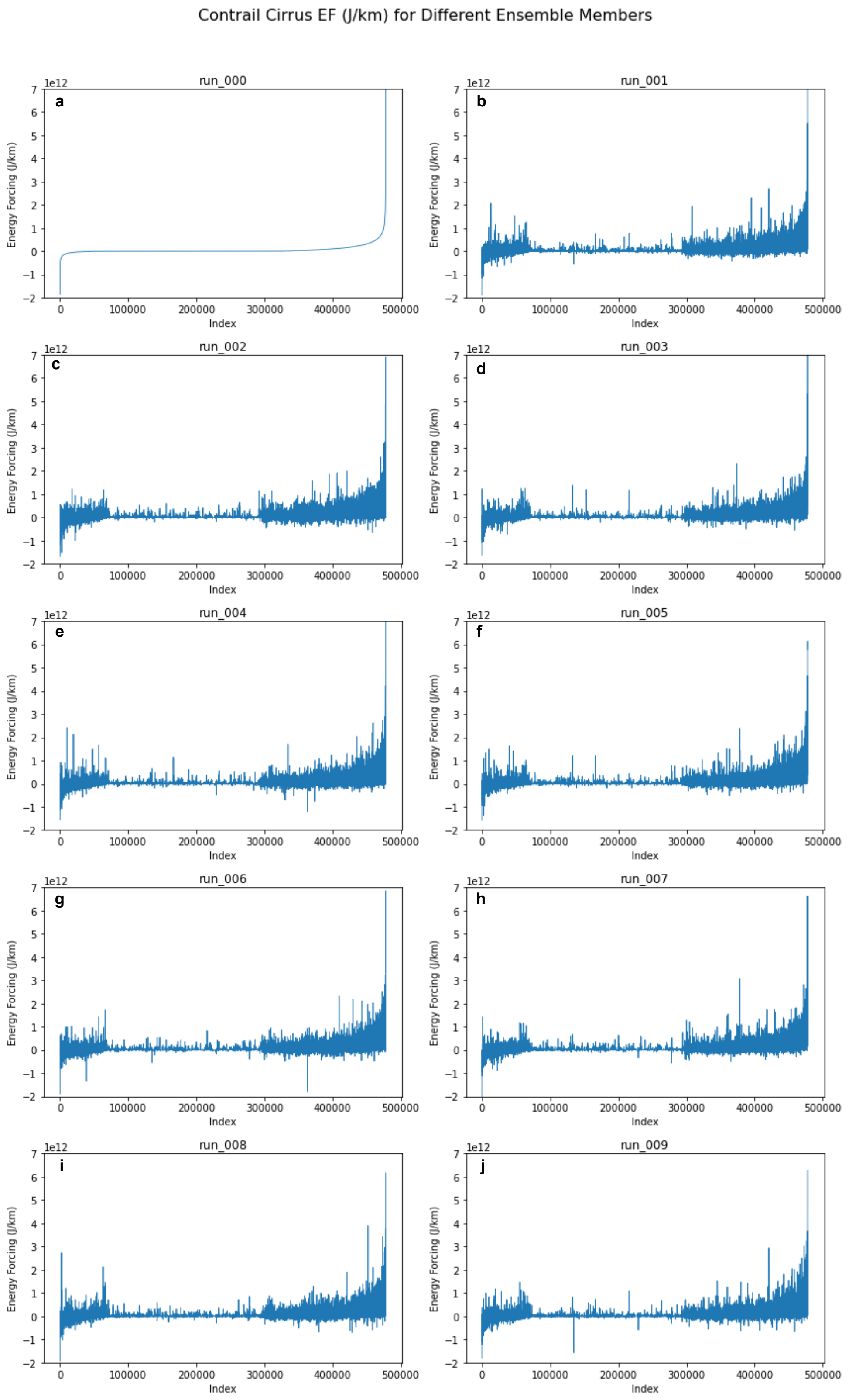

Supplementary Fig. 3. Contrail cirrus energy forcing across ensemble members. Contrail cirrus energy forcing (EF, J/km of flight) for individual flights generated by CoCiP using ten ensemble members of the ERA5 reanalysis as input. Flights are ordered from highest to lowest EF in the control run (run

000; a). The same ordering is applied to the other nine ensemble runs (b–j), each showing the corresponding EF per km of flight.

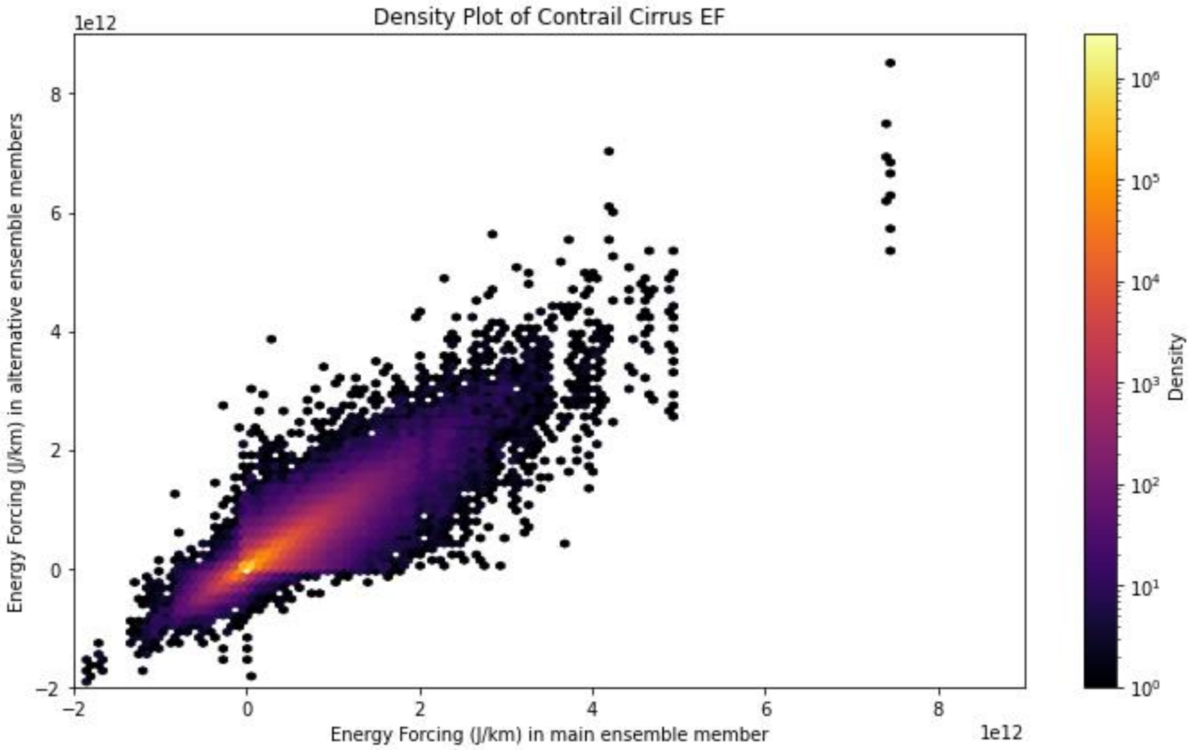

Supplementary Fig. 4. Contrail cirrus energy forcing in control run versus ensemble members. Hexbin plot showing contrail cirrus energy forcing (EF, J/ km) for individual flights generated by CoCiP. The x-axis represents EF from the ERA5 control run, and the y-axis represents EF from the other ensemble members. Color shading indicates the density of observations within each hexagon.

In Supplementary Fig. 5, the number of ensemble members that generate a positive EF for a specific flight is shown. For about half of all flights, CoCiP does not generate warming contrails for any ensemble member, while for about 127 000 flights CoCiP generates warming contrails for all 10 ensemble members. For the remaining approximately 102 000 flights warming contrails are generated in a few, one to nine cases, but not for all cases when using different ERA5 ensemble members as input to CoCiP.

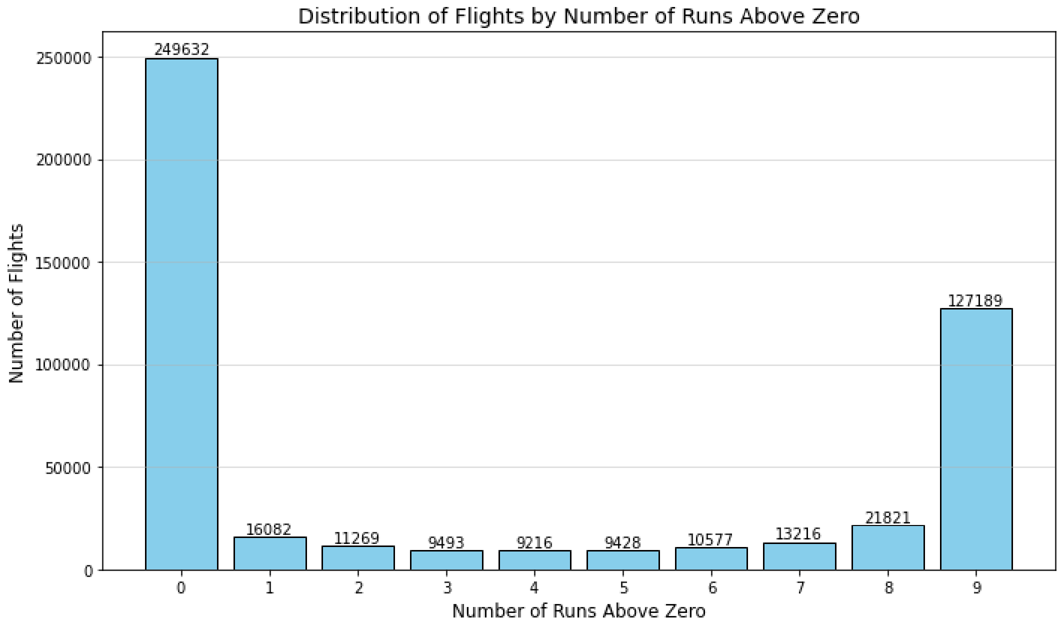

Supplementary Fig. 5. Flights generating positive contrail cirrus energy forcing. Number of flights with positive energy forcing (EF) across different numbers of ensemble member runs. Bar height indicates the number of flights for which a positive EF was generated in a given number of runs.

Supplementary Fig. 6 shows the Probability Density Function (PDF) used for the probabilistic scaling of contrail cirrus EF combined with the efficacy uncertainty. The PDF is used both on the global scale and on a per-flight basis for the CoCiP output in each of the ten different ERA5 ensemble runs.

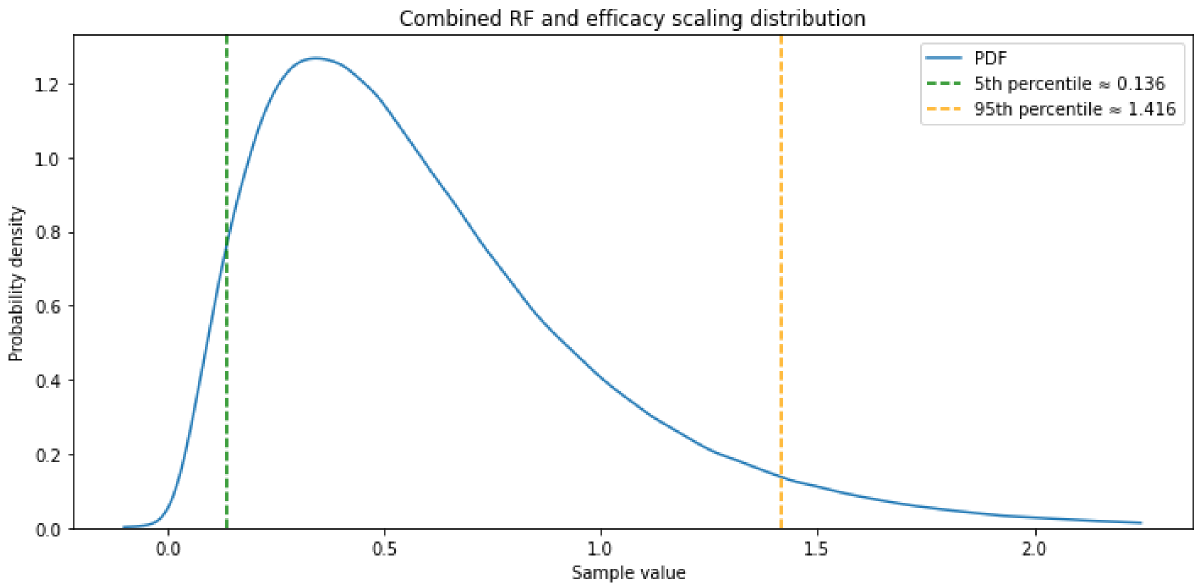

Supplementary Fig. 6. Probability density function for contrail cirrus energy forcing scaling. Probability density function (PDF) used for probabilistic scaling of contrail cirrus energy forcing (EF) to generate efficacy-adjusted EF estimates at the global scale and on a per-flight basis.

Platt et al. (2024) attempts to estimate contrail forcing uncertainty for individual flights[3]. The study considers uncertainties in weather, by using the same 10 ERA5 ensemble members as we do, as well as uncertainties in seven critical parameter assumptions in CoCiP. The paper analyses uncertainty in

contrail formation and energy forcing (EF) by examining 1,000 randomly selected flights every third day during 2019.

We have extracted EF for all flights in Platt et al that operate in the same region of the North Atlantic as the flights considered in our study and compare the uncertainty characteristics to our approach. To be more specific, we compare the uncertainty in EF per flight-kilometre from the flights included in Platt et al[3] to the uncertainty characteristics (in terms of EF per flight-kilometre) of the flights we consider in our paper, when using the 10 different ERA5 ensemble members scaled with probabilistic EF weighting.

In Supplementary Fig. 7, we show the EF per flight-kilometre for the flights with non-zero EF in our probabilistic sample (blue markers), as well as the EF per flight-kilometre for the flights with non-zero EF in Platt et al[3] that pass through the same region. These are plotted against the average contrail cirrus EF caused by the respective flights. The overall pattern of the relationship between average EF per flight-kilometre and its distribution appears relatively similar for our approach and that of Platt et al[3]. However, our approach tends to produce a somewhat wider distribution overall, which is consistent with the fact that we also consider the impact of structural uncertainties, not just parametric uncertainties.

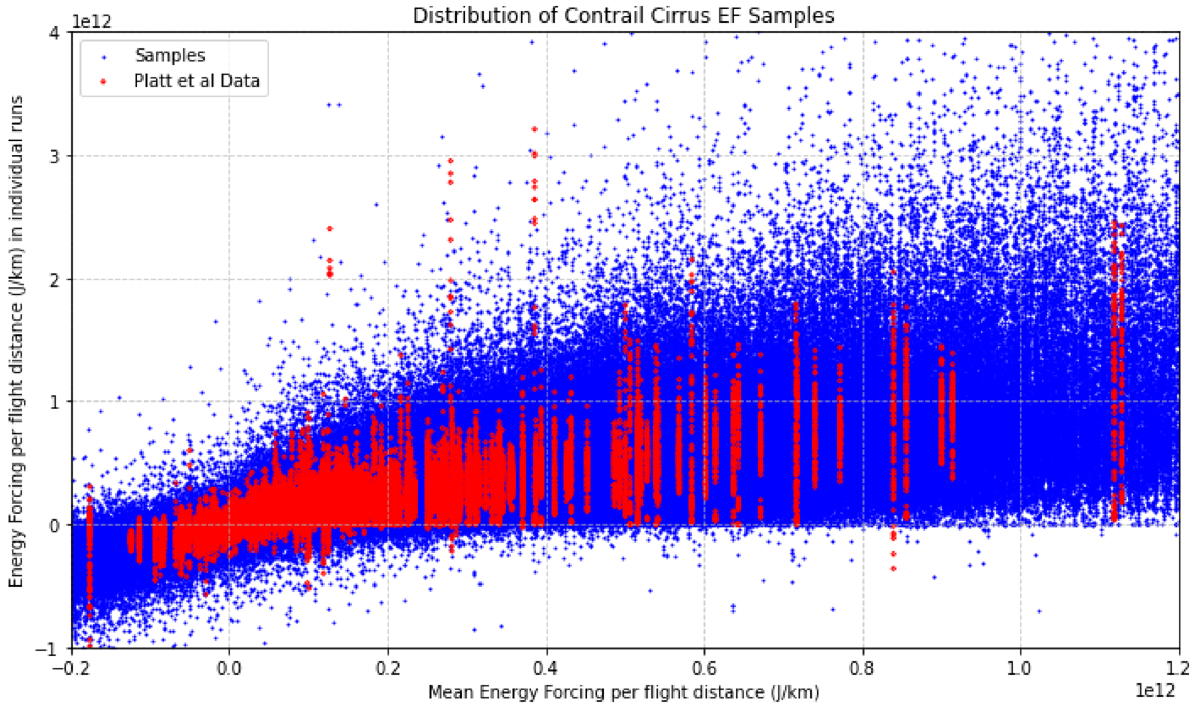

Supplementary Fig. 7. Comparison of probabilistic energy forcing realizations. Probabilistic contrail cirrus energy forcing (EF) realizations plotted against their average values using our approach (blue markers) and the approach by Platt et al. (2024) (red markers).

Supplementary Note 4. Social cost estimates under different assumptions

In Supplementary Table 1 tabulated values for the social costs are presented for different assumptions. The values additional to those presented in the main paper are based on a temperature pathway towards 4°C above the pre-industrial level and one overshoot pathway leading to a stabilization of the temperature at 1°C above the pre-industrial level at 2150 and onwards.

The numbers presented in Supplementary Table 1 can also be compared to earlier estimates of the social cost of contrails. Dorbian et al finds a ratio of SC-contrail to SCC between 0.10 and 3.56 depending on discount rate and contrail cirrus forcing assumptions[4]. The full range obtained using the values in Supplementary Table 1 is between 0.023 and 2.0. One key reason we find a lower maximum value is that the highest discount rate assumed by Dorbian et al is higher than ours.

Grobler et al estimated the SC-contrail as US$ per ton fuel burn, while our estimates Supplemnaty Table 1 are presented as US$ per ton CO_2 emissions[5]. Converting the estimates by Grobler et al gives a range between 3.2 and 73 US$ per ton CO_2 emissions (if assuming 3.16 kg CO_2 per kg fuel burn). Our full range in table SI1 is 4.9 to 550 US$ per ton CO_2 emissions. A key reason why our upper estimate is so much higher than the upper estimate in Grobler et al is the use of the H & S damage function, and the low discount rate in our low case.

Supplementary Table 1. The social costs of CO_2 (SCC), of Energy Forcing (SCEF), and of contrail cirrus (SC-contrail). The values are based on different assumptions on contrail forcing (the main estimate based on Teoh et al (2024)[6] and the 5 and 95 percentiles of the efficacy adjusted contrail cirrus energy forcing), the damage function (being either Howard & Sterner (2017)[7] (H & S) or Nordhaus (2018)[8]), for three different discount rates and for five different temperature stabilization levels (ΔT). The social cost of contrail is normalized to the aviation CO_2 emissions, i.e., based on the global contrail cirrus efficacy adjusted EF per ton emitted CO_2 from aviation.

Damage function	Discount rate	ΔT	SCC (US$/ton$CO_2$)	SCEF (US$/GJ)	SC-contrail (US$/ton$CO_2$)	SC-contrail 5% (US$/ton$CO_2$)	SC-contrail 95% (US$/ton$CO_2$)
H & S	*Low*	*4*	2300	0.34	160	53	550
H & S	*Low*	*3*	1800	0.29	140	44	460
H & S	*Low*	*2*	1200	0.22	100	34	350
H & S	*Low*	*1.5*	910	0.19	88	29	300
H & S	*Low*	*1.0*	620	0.16	76	25	260
H & S	*Medium*	*4*	870	0.21	99	32	330
H & S	*Medium*	*3*	710	0.19	88	29	300
H & S	*Medium*	*2*	510	0.16	74	24	250
H & S	*Medium*	*1.5*	400	0.14	67	22	230
H & S	*Medium*	*1.0*	310	0.13	63	20	210
H & S	*High*	*4*	120	0.11	52	17	170
H & S	*High*	*3*	110	0.10	51	16	170
H & S	*High*	*2*	93	0.10	49	16	160
H & S	*High*	*1.5*	84	0.10	48	15	160
H & S	*High*	*1*	80	0.10	47	15	160
Nordhaus	*Low*	*4*	640	0.10	47	15	160
Nordhaus	*Low*	*3*	510	0.084	40	13	130
Nordhaus	*Low*	*2*	360	0.066	31	10	110
Nordhaus	*Low*	*1.5*	270	0.057	27	8.7	91
Nordhaus	*Low*	*1*	190	0.050	24	7.7	80
Nordhaus	*Medium*	*4*	250	0.063	30	10	101
Nordhaus	*Medium*	*3*	210	0.057	27	8.7	91
Nordhaus	*Medium*	*2*	150	0.049	23	7.5	78

Nordhaus	*Medium*	*1.5*	120	0.044	21	6.8	71
Nordhaus	*Medium*	*1*	96	0.042	20	6.4	67
Nordhaus	*High*	*4*	36	0.034	16	5.3	55
Nordhaus	*High*	*3*	33	0.033	16	5.1	53
Nordhaus	*High*	*2*	29	0.032	15	5.0	52
Nordhaus	*High*	*1.5*	26	0.032	15	4.9	51
Nordhaus	*High*	*1*	25	0.032	15	4.9	51

References

1 Riahi, K. *et al.* Mitigation Pathways Compatible with Long-term Goals (Chapter 3). (2022).

2 Hersbach, H. *et al.* The ERA5 global reanalysis. *Quarterly journal of the royal meteorological society* **146**, 1999-2049 (2020).

3 Platt, J. C. *et al.* The effect of uncertainty in humidity and model parameters on the prediction of contrail energy forcing. *Environmental Research Communications* **6**, 095015 (2024).

4 Dorbian, C. S., Wolfe, P. J. & Waitz, I. A. Estimating the climate and air quality benefits of aviation fuel and emissions reductions. *Atmospheric Environment* **45**, 2750-2759 (2011).

5 Grobler, C. *et al.* Marginal climate and air quality costs of aviation emissions. *Environmental Research Letters* **14**, 114031 (2019).

6 Teoh, R. *et al.* Global aviation contrail climate effects from 2019 to 2021. *Atmospheric Chemistry and Physics* **24**, 6071-6093 (2024).

7 Howard, P. H. & Sterner, T. Few and not so far between: a meta-analysis of climate damage estimates. *Environmental and Resource Economics* **68**, 197-225 (2017).

8 Nordhaus, W. Projections and uncertainties about climate change in an era of minimal climate policies. *American economic journal: economic policy* **10**, 333-360 (2018).
